# Radiofrequency ablation-induced superior vena cava stenosis in a 5-year-old boy with congenital left atrial appendage deformity: a case report and literature review

**DOI:** 10.3389/fsurg.2023.1199335

**Published:** 2023-07-10

**Authors:** Jianxian Xiong, Yu Wenbo, Jianfeng Gao, Meifang Li, Dongmin Yu

**Affiliations:** ^1^ Department of Cardiovascular Surgery, First Affiliated Hospital of Gannan Medical University, Ganzhou, China; ^2^ The First Clinical Medical College, Gannan Medical University, Ganzhou, China; ^3^ Department of Breast Disease Comprehensive Center, First Affiliated Hospital of Gannan Medical University, Ganzhou, China

**Keywords:** superior vena cava, left atrial appendage aneurysm, radiofrequency ablation, arrhythmias, complication

## Abstract

Superior vena cava (SVC) stenosis is rarely caused by iatrogenic trauma. Herein, the case of a 5-year-old boy who underwent radiofrequency ablation for paroxysmal supraventricular tachycardia but developed SVC stenosis and related syndromes is reported. Notably, the child exhibited an enlarged left atrial appendage that had partially breached the pericardium. Subsequent interventions involved successful removal of the stenosis, artificial vascular reconstruction, and comprehensive radiofrequency ablation of the entire right atrium, along with ligation of the left atrial appendage under direct vision. As a result, the child experienced relief from symptoms.

## Background

Superior vena cava (SVC) stenosis is a critical condition characterised by SVC compression by intrathoracic masses and/or endovascular devices (1). While a recent study suggests that intraluminal tumour metastasis can also cause SVC stenosis (2), it is rarely observed as a result of radiofrequency ablation. Surgical interventions are typically required to address SVC stenosis and SVC syndrome by eliminating the obstruction. In cases where the obstruction is caused by intraluminal tumour metastasis, radiotherapy and chemotherapy are also feasible. Without intervention, oedema might compress the brain, midbrain, and medulla oblongata, resulting in acute syndromes, like hernia, loss of consciousness, and respiratory arrest (3). An enlarged left atrial appendage, known as left atrial appendage aneurysm (LAAA), is a rare condition that can be classified as intrapericardial or extrapericardial LAAA based on pericardial integrity or as congenital and secondary LAAA based on the underlying causes (4–6). Secondary left auricular enlargement can result from severe mitral stenosis or reflux, which increases left atrial pressure. Congenital LAAA might arise from congenital defects in the atrial comb muscle, particularly when the atrium expands due to blood flow and intracardiac pressure (6). Tumours with an enlarged left atrial appendage can compress the ventricles and cause cardiac insufficiency. Additionally, the presence of thrombi in the left atrial appendage could result in systemic circulation embolism and malignant arrhythmias (7). Therefore, early treatment is crucial once these conditions are diagnosed. Herein, a case of artificial vascular replacement of SVC stenosis in a 5-year-old boy with LAAA is reported and a concise literature review on SVC stenosis and LAAA is provided (Table 1) (8–19). Common surgical interventions for SVC stenosis include SVC angioplasty, percutaneous transluminal angioplasty, and SVC stenting. However, there have been no previous reports of vascular replacement in a 5-year-old child.

**Table 1:** Etiologies and therapies of SVC stenosis or LAAA in case series.

Citation	Etiology of SVC stenosis or LAAA	Number of patients	Treatment (*n*)
SVC stenosis			
Kuhne et al. (13)	Mediastinitis,compressed or involved by the tumors	22	Gianturco-Rösch expandable Z-stents (22)
McDevitt et al. (20)	Haemodialysis	11	PTA (3), PTA and lysis (2), PTA and superior vena Cava stenting (2), Conservative (4)
Nishiyama et al. (21)	Congenital	1	Pericardial patch augmentation (1)
Gaita et al. (22)	Lipomatosis of the right atrium	1	Surgical resection (1)
Thomas et al. (23)	BioGlue complication	1	Balloon dilatation and stent placement (1)
Jin et al. (24)	Radiofrequency ablation	1	Repeat ablation (1)
LAAA			
Zhang et al. (25)	Congenital and acquired conditions	5	Surgical resection (1), drug management (1), patient refusal (1), Waiting for surgery (1), loss to follow-up (1)
Jiang et al. (26); Stanford et al. (27)	Congenital	2	Conservative (2)
Yakut et al. (18); Parmley et al. (28)	Congenital	2	Surgical resection (2)
Aryal et al. (29)	Congenital	1	Anticoagulation management and surgery (1)
Quaretti et al. (30)	Hurler-Scheie syndrome	1	Urgent Surgical resection (1)

PTA, percutaneoius transluminal angioplasty.

## Case presentation

**Figure 1:**
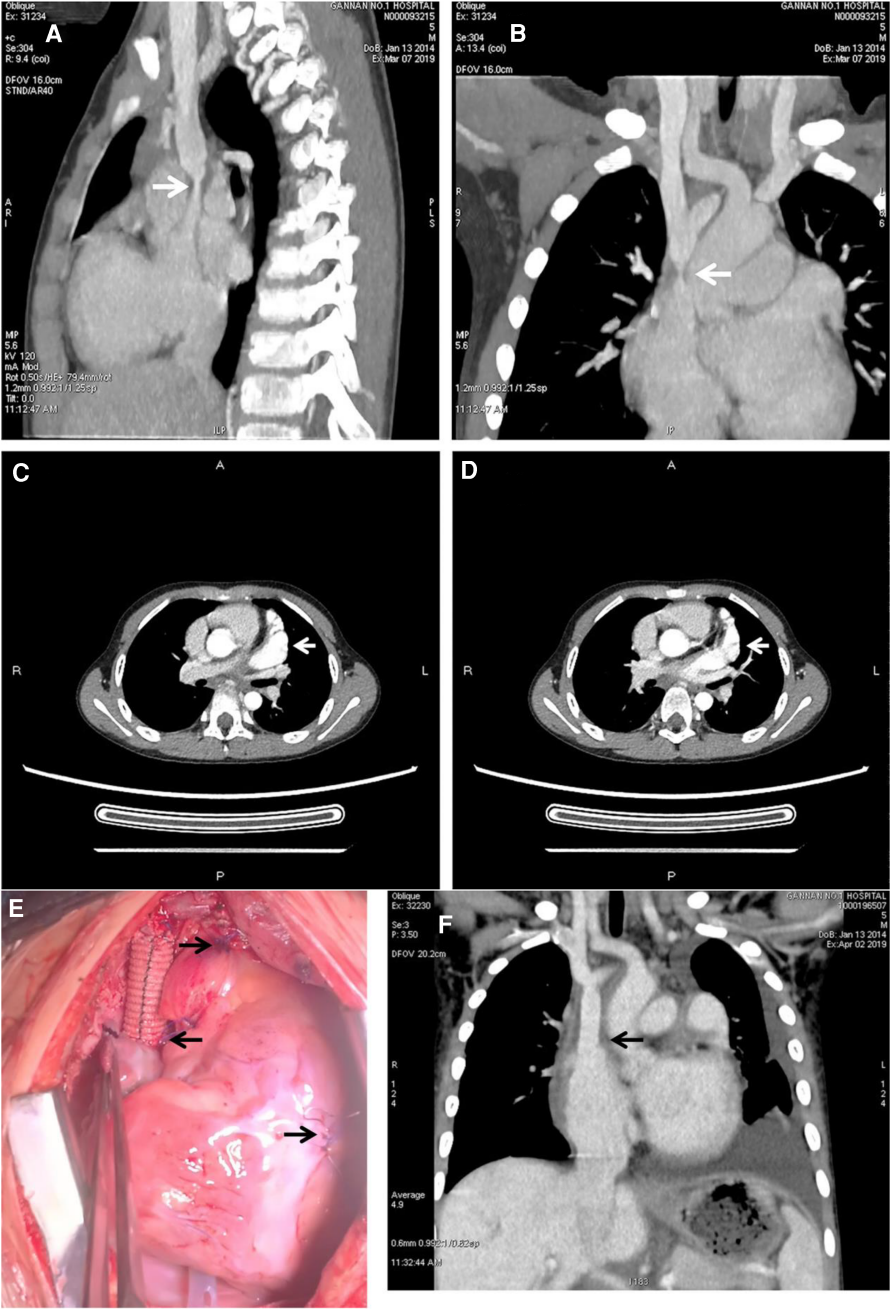
Preoperative CT and CTA showed the stenosis of the superior vena cava and left atrial appendage aneurysm. Intraoperative and postoperative CT showed the reconstruction of the superior vena cava with artificial blood vessels. (**A,B**) Showed severe stenosis at the junction of superior vena cava and right atrium (arrowhead). (**C,D**) Showed that the enhancement of the left atrial appendage was consistent with that of the ascending aortic cavity, and the arrowhead referred to the enlarged left atrial appendage (arrowhead). (**E**) Showed the details of the operation; the left arrow showed the site of anastomosis between the artificial vessel and the right atrium; the first arrow on the right showed the site of ligation and suture at the root of the left atrial appendage; and the second arrow on the right showed the site of the temporary pacemaker. (**F**) Showed that the blood flow between the artificial vessel and the right atrium was unobstructed, which relieved the stenosis of the superior vena cava.

**Figure 2:**
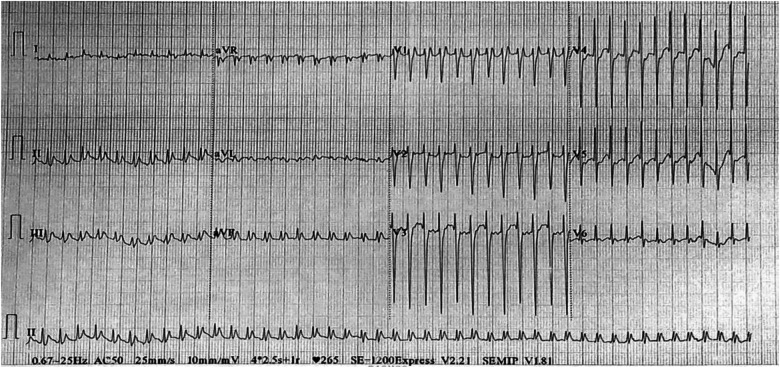
Electrocardiography showed paroxysmal supraventricular tachycardia.

A 5-year-old boy was hospitalised due to facial and upper limb oedema. He was diagnosed with paroxysmal supraventricular tachycardia at another hospital a month ago. Prior to admission, prehospital electrophysiological (EP) testing and electroanatomical mapping had confirmed the ectopic stimulation point in the SVC. Subsequently, the patient underwent catheter ablation using a temperature-controlled mode, with the target temperature set at 50°C and energy at 30 Watts. However, a month after the procedure, the child developed facial and upper limb oedema, complicated by headache and mental disorder. Furthermore, episodes of supraventricular tachycardia persisted. Upon admission to our hospital, computed tomography (CT) angiography (CTA) revealed SVC stenosis (Figures 1A,B). Contrast-enhanced CT revealed an enlarged left atrial appendage (Figures 1C,D). Electrocardiogram findings indicated paroxysmal supraventricular tachycardia (Figure 2). Esmolol was administered to control the heart rate.

After obtaining informed consent, the patient underwent surgery. Ascending aortic cannulation was performed, and an SVC drainage tube was inserted at the confluence of the innominate vein and the SVC. Additionally, an inferior vena cava drainage tube was inserted through the right atrium. Cold blood cardioplegia solution was infused to protect the myocardium. Intermittent anterograde delivery of cold blood cardioplegia solution was performed via the aortic root during normothermic cardiopulmonary bypass. The initial plan was to use a patch to expand the narrowed segment of the SVC. However, upon opening the SVC, severe vascular wall damage and fragile scar tissue that could not support the patch were observed. Therefore, a 10 mm artificial vessel was used to replace the SVC. The stenosis was excised, and the SVC was reconstructed. Special care was taken to safeguard the sinoatrial node during suturing. Subsequently, the patient underwent a maze procedure using surgical ablation. The Medtronic bipolar radiofrequency ablation system (Medtronic, Minneapolis, MN, USA) was employed to perform radiofrequency ablation of the entire right atrium (including the right atrial appendage, right atrial wall, atrial septum, and tricuspid isthmus) to isolate ectopic rhythms or re-entrant activations originating from the right atrium. Furthermore, ligation of the left atrial appendage was performed to prevent the formation of thrombi or thromboembolism, such as cerebral embolism and arterial embolism, resulting from LAAA. Upon restoration of heart function, radiofrequency ablation demonstrated satisfactory efficacy.

**Figure 3:**
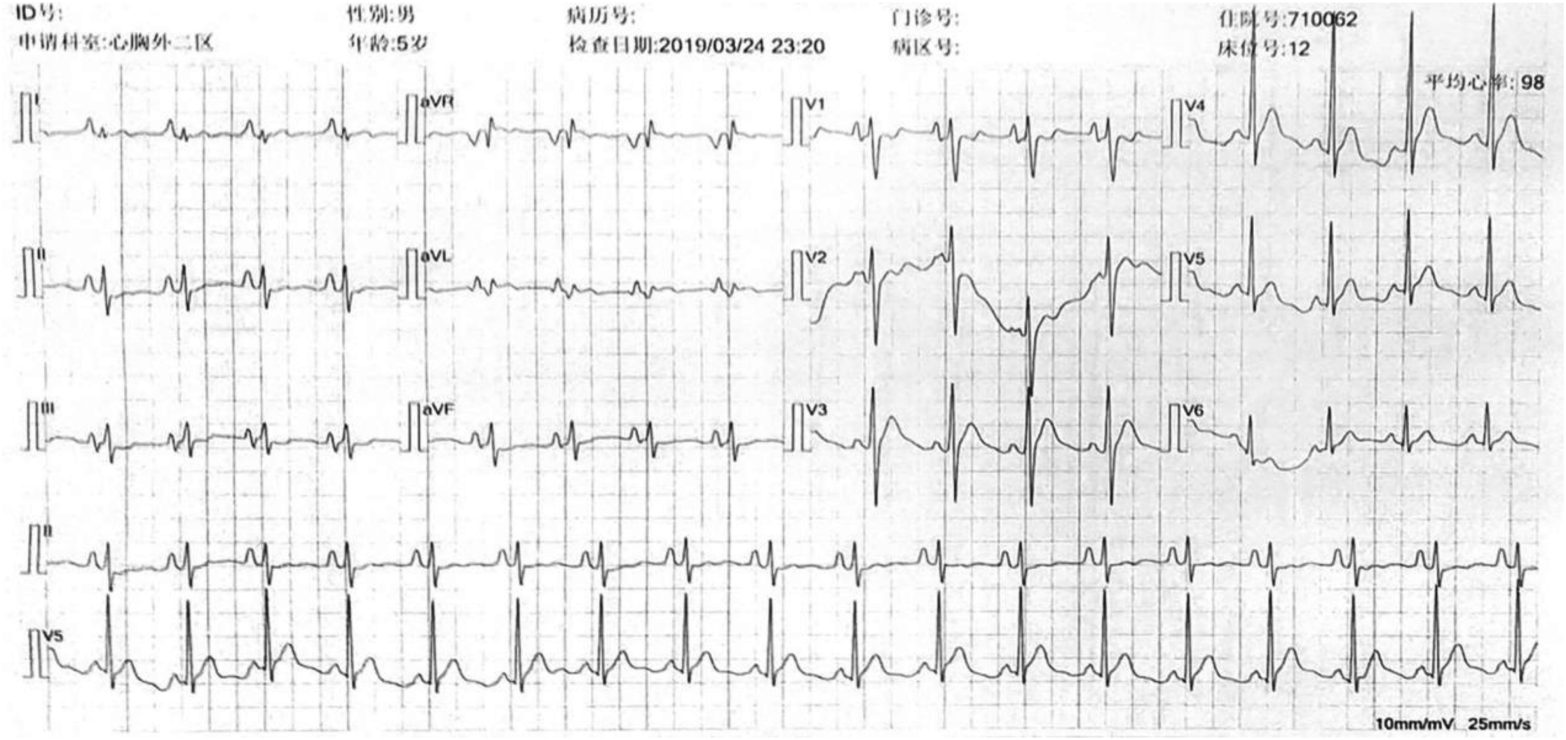
Preoperative ECG shows that the heart rhythm was paroxysmal supraventricular tachycardia.

Following the surgery, the patient's facial and upper extremity oedema subsided. CTA revealed that the SVC regained vascular patency (Figure 1F), and the electrocardiogram showed a recovered sinus rhythm (Figure 3). Subsequently, the patient was discharged and prescribed aspirin for 3 months. Telephonic follow-up assessments were performed at 3 months and 6 months after discharge, during which the patient remained asymptomatic without any adverse events.

## Discussion

SVC syndrome is a complex condition primarily characterised by severe stenosis or obstruction of the SVC. The degree of obstruction can be categorised into four types (Table 2) (31). In 1997, Lumsden et al. (32) proposed diagnostic criteria for severe SVC stenosis, defining it as a stenosis with a diameter reduction of >50% in the SVC, with or without upstream vein stenosis. These criteria have been widely adopted in the field of veno-occlusive diseases. Clinical symptoms associated with severe SVC stenosis include facial, neck, and/or upper chest oedema, which can extend to the proximal end of the upper limbs. Venography could be used to assess the presence and severity of SVC stenosis. Besides the congenital causes, SVC stenosis is primarily attributed to internal or external factors, including long-term indwelling central venous catheter (CVC), transvenous pacemakers, surgeries, and radiotherapy. Prolonged exposure to these factors stimulates vascular endothelial cells, leading to intimal inflammation, intimal hyperplasia, and eventually SVC stenosis (30). The most common cause is long-term CVC retention in patients with end-stage renal disease undergoing haemodialysis (27, 33). External factors contributing to SVC stenosis often involve compression by mediastinal masses, malignant tumours, or fibrous mediastinitis. The SVC, located in the middle mediastinum, is surrounded by mediastinal fat, lymph nodes, the right lung and pleura, and the left trachea and ascending aorta. Enlargement of these structures might compress the SVC. Malignant tumours, including small cell and non-small cell lung carcinoma, lymphoma, metastatic lymphadenopathy from intrathoracic and extrathoracic malignant tumours, and tracheal malignant tumours, are the most common external factors causing SVC stenosis (8, 20). Chest CT is the most common imaging technique, while magnetic resonance (MR) angiography can provide better visualisation of cancer cell infiltration in blood vessels and pericardium. The main approach for addressing stenosis caused by internal factors is removing the stimulating factor, such as removing CVC or pacemaker leads. If infection and thrombosis occur, symptomatic support treatments such as antibiotics and anticoagulation might be necessary. In cases of stenosis due to external factors, relieving the compression becomes essential. If the compression cannot be relieved, venous angioplasty and stenting could be considered to improve patient symptoms.

**Table 2:** Types of superior vena cava obstruction.

Type	Degree of SVC obstruction	Feature
I	Partial obstruction (<90%)	With azygos vein and right atrial pathway open
II	Almost complete obstruction (>90%)	With the anterograde direction of the azygos vein, it flows to the right atrium.
III	Almost complete obstruction (>90%)	With azygos venous blood reflux.
IV	Complete obstruction	Complete occlusion of vena cava and azygos vein.

**Figure 4:**
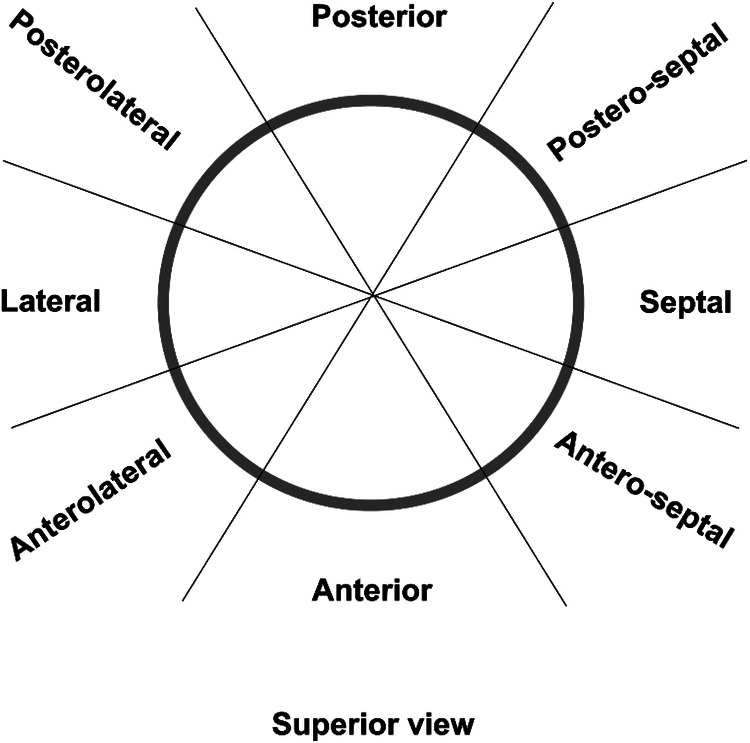
Distribution of the ablation points at the superior vena cava-right atrium (SVC-RA) junction. Superior view of the SVC-RA junction is shown.

In this case, the patient exhibited progressive facial oedema and developed headaches one week after undergoing radiofrequency ablation. CTA confirmed the presence of severe local SVC stenosis, which was believed to be caused by SVC injury after radiofrequency ablation. Urgent removal of the SVC obstruction was necessary to prevent complications such as cerebral oedema, cerebral compression, cerebral herniation, and acute compression syndromes affecting the midbrain and medulla, resulting in loss of consciousness, respiratory arrest, and other complications (3). The width of the artificial vessel was chosen to suit the age of the child, considering that the patient was too young and that an artificial vessel that was too thick might interfere with cranial blood flow. Therefore, in this patient, a 10 mm artificial vessel was used for SVC replacement. Following the surgery, the patient's facial and upper extremity oedema subsided. And it is essential to monitor the biological adaptability of the artificial vessel and assess whether further surgical intervention will be required. Cardiac radiofrequency ablation is the most effective method for treating paroxysmal tachycardia. By delivering radiofrequency current through the electrode, it induces myocardial coagulative necrosis, thereby blocking the abnormal conduction bundle and subsequent tachyarrhythmia. Precisely identifying the location of the sinoatrial node is crucial for cardiac radiofrequency ablation as it determines the ablation approach and significantly affects patient prognosis. The distribution of the sinoatrial node is primarily based on two parameters: height distribution and plane distribution. Height distribution involves three locations: the SVC, right atrium, and SVC-right atrial junction. The sinoatrial node is considered to be in the right atrium when its height is two-thirds lower than the SVC-right atrial junction, and in the SVC when its height is two-thirds higher than the SVC-right atrial junction (21). Plane distribution is described by the head and foot position, 1–3 o’clock position corresponds to the postero-septal position, 3–5 o’clock to the antero-septal position, 5–7 to the anterior position, 7–9 o’clock to the anterolateral position, 9–11 o’clock to the posterolateral position, and 11–1 o’clock to the posterior position (Figure 4). In cases of paroxysmal supraventricular tachycardia originating from the SVC, ablation can be performed on the septal side of the SVC-right atrium without cardioversion. However, if the sinoatrial node is located within the superior vena cava or the ablation target in the SVC overlaps with the functional sinoatrial node, the ablation level on the side of the sinoatrial node should be at least 5 mm higher than that of the sinoatrial node. This approach ensures the maximum protection of the superior vena cava from injury, which is particularly crucial in paediatric patients whose SVC walls are not fully developed.

Typically, radiofrequency currents can ablate an area within a radius of 1–3 mm. Supraventricular tachycardia often presents with sudden attacks, palpitation, shortness of breath, chest tightness, dizziness, and lethal syncope or shock (22). For patients experiencing recurrent episodes, endocardial EP examination and radiofrequency ablation are recommended. Transcatheter ablation is safe and effective for children; however, those under 4 years of age and weighing <15 kg have a higher likelihood of experiencing complications (23). The most frequently observed complications of radiofrequency catheter ablation are sinoatrial node injury, phrenic nerve paralysis, and SVC stenosis (24, 25). Given that the cardiovascular system of children is still maturing, radiofrequency ablation might result in vascular damage and pericardial tamponade.

LAAA is a rare abnormality with potentially fatal complications (26). The size of LAAA can range from 4 cm × 3 cm to 22 cm × 15 cm, with an average size of 11 ± 5 × 7 ± 3 cm. This first reported case of LAA was documented by Parmley in 1962 (28). While LAAA can occur in individuals of all age groups, it is most commonly observed in patients aged approximately 30 years old, with an average age of 30 ± 20 years (16). The incidence of LAAA tends to increase with age. As the aneurysm grows larger, patients might experience symptoms such as supraventricular arrhythmia, cardiac insufficiency, atypical chest pain, and an elevated risk of intra-atrial thrombosis and systemic embolism (29). Several reports have linked LAAA to atrial or supraventricular arrhythmias (7, 34). Among the patients, 27 (26.7%) patients exhibited atrial fibrillation/flutter, 10 (9.9%) had supraventricular tachycardia, and 60 (59.4%) maintained sinus rhythm. The elevated tension exerted by LAAA on the conduction system or congenital defects in the conduction tissues might contribute to the occurrence of supraventricular arrhythmias (16). Therefore, in young patients presenting with atrial fibrillation without other associated cardiac abnormalities, it is crucial to consider the possibility of LAAA. The diagnosis of LAA can be confirmed using transthoracic echocardiography or transoesophageal echocardiography with colour Doppler, which can reveal blood flow between the left atrium and LAAA (35). Other imaging modalities, such as CT, MR imaging, CTA, and angiocardiography, are valuable in diagnosing LAAA and ruling out other conditions, such as cardiac or mediastinal tumours, pericardial cysts, acquired left atrial enlargement secondary to mitral valve disease, and left atrial hernia with pericardial defects (19, 36, 37). Once diagnosed, surgery is the recommended treatment option, even in asymptomatic patients, as it can prevent potential thromboembolism and address associated arrhythmias. The two most commonly used techniques are cardiopulmonary bypass-assisted median sternotomy and left lateral sternotomy. The former is employed in patients with left atrial thrombus, as cardiopulmonary bypass blocking the aorta could prevent systemic embolism during aneurysm resection. On the other hand, left lateral sternotomy offers better visualisation and causes less patient trauma compared with median sternotomy.

In this case report, median sternotomy aided with cardiopulmonary bypass was employed due to the requirement for angioplasty. During the surgery, a pericardial defect surrounding the left atrial appendage was incidentally discovered, necessitating an extrapericardial repair. An enlarged left atrial appendage can compress the ventricles, resulting in cardiac insufficiency, while sluggish blood flow within the left atrial appendage could contribute to thrombus formation and subsequent systemic circulation embolism. Therefore, early intervention is necessary. In this case, prehospital EP testing and electroanatomical mapping confirmed the ectopic stimulation point was located at the SVC. Subsequently, radiofrequency ablation of the right SVC was performed immediately after diagnosis. Although the arrhythmia symptoms subsided within a month, they reappeared later with increased frequency. This suggests the possibility of multiple tachycardia origin points or incomplete success of the previous interventional radiofrequency ablation. Therefore, radiofrequency ablation was performed on the entire right atrium to avoid the recurrence of arrhythmia. Additionally, considering the patient's young age (5 years), ligation of the left atrial appendage was performed to prevent thrombus formation or thromboembolism, such as cerebral embolism and arterial embolism, resulting from LAAA.

## Conclusions

Continuous documentation of these rare cases is important for guiding future practices. This case stands apart from previous cases in that the SVC syndrome resulted from radiofrequency ablation, and the patient is a 5-year-old boy with congenital LAAA. Therefore, for children with arrhythmia and LAAA, surgical intervention might offer a more favourable option than radiofrequency ablation. Simultaneous to the surgical intervention, surgical ablation can be performed to achieve complete resolution of LAAA and arrhythmia. Additionally, for children with irreparable superior vena cava stenosis, artificial vascular replacement serves as a potential alternative. Nonetheless, it is important to note that the long-term prognosis of children in such cases warrants ongoing observation and follow-up.

## Data Availability

The original contributions presented in the study are included in the article, further inquiries can be directed to the corresponding author.
